# Simplified screening approach of anabolic steroid esters using a compact atmospheric solid analysis probe mass spectrometric system

**DOI:** 10.1007/s00216-022-03967-y

**Published:** 2022-02-27

**Authors:** Ane Arrizabalaga-Larrañaga, Paul W. Zoontjes, Johan J. P. Lasaroms, Michel W. F. Nielen, Marco H. Blokland

**Affiliations:** 1 Department of Chemical Engineering and Analytical Chemistry, University of Barcelona, Av. Diagonal 645, 08028 Barcelona, Spain; 2 Wageningen Food Safety Research (WFSR), Part of Wageningen University & Research, P.O. Box 230, 6700 AE Wageningen, The Netherlands; 3 Laboratory of Organic Chemistry, Wageningen University, Stippeneng 4, 6708 WE Wageningen, The Netherlands

**Keywords:** Mass spectrometry, Ambient ionization, On-site testing, Atmospheric solids analysis probe, Transportable MS

## Abstract

Due to the absence of chromatographic separation, ambient ionization mass spectrometry had the potential to improve the throughput of control laboratories in the last decades and will soon be an excellent approach for on-site use as well. In this study, an atmospheric solids analysis probe (ASAP) with a single quadrupole mass analyzer has been evaluated to identify anabolic steroid esters rapidly. Sample introduction, applied scan time, and probe temperature were optimized for sensitivity. The in-source fragmentations of seventeen selected steroid esters, commonly found in illicit samples, were determined by applying different cone voltages (12, 20, 30, and 40 V). A spectral library was created for these steroid esters based on the four stages of in-source fragmentation spectra. The applicability of this method was demonstrated for the rapid identification of steroid esters in oily injection solutions, providing test results in less than 2 min.

**Graphical abstract:**

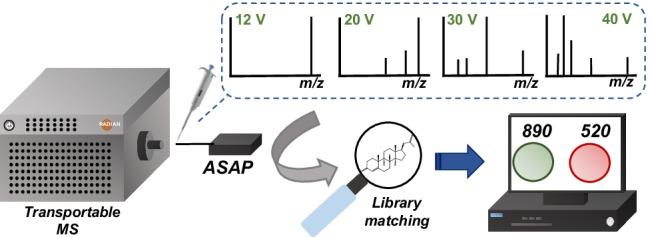

**Supplementary Information:**

The online version contains supplementary material available at 10.1007/s00216-022-03967-y.

## Introduction

The use of ambient ionization mass spectrometry (AIMS) in food and forensic control laboratories to improve their laboratory throughput has increased during the last decade [1, 2]. This increase is due to the vast number of AIMS techniques available nowadays [3]. AIMS techniques allow rapid, real-time, high-throughput, and in situ analysis of solids, liquids, and gases and are characterized by their simplified sample preparation and sample introduction protocols, reduced costs, and analysis time. Some of them, such as desorption electrospray ionization (DESI) [4], direct analysis in real-time (DART) [5], laser ablation electrospray ionization (LAESI) [6], and atmospheric solids analysis probe (ASAP) [7], are commercially available. However, they are mostly used in specialized laboratories or for research purposes, and their potential for future on-site testing is not well established yet. The development of portable and miniaturized mass spectrometers will significantly support the development of on-site chemical analysis. AIMS techniques such as DESI, DART, and low temperature plasma (LTP) ionization, among others, have already been reported as potential on-site testing methods for chemicals in the field of food and forensics [8–17].

Among AIMS techniques, ASAP has shown great potential for rapidly detecting residues, contaminants, and adulterants in food control [18]. The ASAP technique was introduced in 2005 by McEwen et al. [7] for the fast direct analysis of volatile and semi-volatile, solid and liquid samples under atmospheric pressure conditions. For instance, Cechova et al. [19] evaluated the direct surface analysis of pea seeds for the study of fatty acids profile and Doue et al. [20] analyzed steroid esters in oily injection preparations. Steroid esters are commonly used to enhance performances and improve the growth rate of both humans and animals, with the potential for misuse as growth promoters in animal husbandry and are banned in the European Union for the use of cattle fattening [21]. For official control, laboratories and veterinary, inspectors, and authorities present an easy-to-use, difficulties for the fast and reliable detection of these illicit substances in oily injection preparations for veterinary use would make the control of samples more efficient. For this purpose, the determination of steroid esters in the study that Doue et al. [20] developed was carried out by an ASAP–MS/MS method using a benchtop triple quadrupole mass analyzer. Although the described approach is useful to support the control and enforcement of authorities, it is not suitable for future on-site analysis at, for example, border inspection points or slaughterhouses and lacks automated software interpretation of the data. Thus, each spectrum had to be assessed on itself, and non-expert users could not easily interpret the obtained results. A new commercially available instrument was recently introduced that combined ASAP and a single quadrupole transportable MS system. This new instrument also includes an informatics solution LiveID 2.0 package that addresses the needs of complex analytical challenges such as food authenticity, offering real-time classification of samples either through chemical spectra fingerprint or mass spectral library matching. Non-expert users can use the interface as results are displayed using a green or red traffic light system that will tell the end-user if a sample contains any undesired contaminants. In addition, it has shown great potential for the high-throughput authenticity screening of commercial herbs with multivariate analysis [22].

In this study, the potential of the compact ASAP–MS system for fast screening and identification of steroid esters in oily preparations is assessed. To this end, the ASAP–MS was optimized, and the most critical working parameters were evaluated and discussed. A library was created for the real-time library matching of steroid esters in real samples based on their in-source fragmentation mass spectra. The applicability of the ASAP–MS technique has been demonstrated by identifying suspect compounds in injection preparations based on match score.

## Experimental section

### Chemicals and materials

Formic acid (≥ 98%), LC–MS grade acetonitrile, methanol, and water were purchased from Actu-All Chemicals (Oss, the Netherlands). Ammonia solution 25%, acetic acid (glacial 100%), acetone for pesticide residue analysis (≥ 99%), and toluene for HPLC (≥ 99%) were supplied from Merck (Darmstadt, Germany). Analytical standards of seventeen steroids as representative of the most common on websites specialized in the anabolic steroids black market, including testosterone acetate (T Ac), testosterone propionate (T Pr), testosterone isocaproate (T Iso), testosterone enanthate (T En), testosterone decanoate (T Dc), testosterone benzoate (T Bz), testosterone phenylpropionate (T PhPr), testosterone cypionate (T Cy), Nortestosterone phenylpropionate (N PhPr), boldenone undecylenate (B Un), estradiol dipropionate (E2 DiPr), estradiol valerate (E2 V1), estradiol benzoate (E2 Bz), trenbolone (Tr), trenbolone acetate (Tr Ac), trenbolone enanthate (Tr En), and drostanolone enanthate (D En), were purchased from Steraloids Inc. Ltd. (London, England). Individual stock solutions (1,000 mg L^−1^) were prepared in methanol and stored at − 80 °C. Intermediate individual solutions (10 mg L^−1^) and a standard mixture solution (1 mg L^−1^) containing all target compounds were prepared monthly from stock standard solution by appropriate dilution in acetonitrile:water (50:50, *v/v*). All standard solutions were stored at 4 °C until their use.

### Instrumentation

The study was carried out on a RADIAN ASAP instrument based on the model QDa (single quadrupole) mass analyzer and equipped with a horizontal loading fixed geometry ASAP source (Waters Corporation, Manchester, UK). Sealed glass capillaries (1.9 mm diameter) were used for sampling via spotting liquid extracts (2 μL) onto the capillary tip. Each capillary was inserted into the ASAP source prior to the sample loading to remove any possible contaminants from the capillary surface and reduce the background noise of the spectra by applying the “bake-out” step, which consists of exposing the capillary to nitrogen gas at high temperature (circa 600 °C) for 60 s. The ASAP–MS optimal working conditions were as follows: nitrogen gas flow and isothermal temperature heating were 3.0 L min^−1^ and 450 °C, respectively, and probe temperature was 150 °C. Besides, the corona current was set at + 3 μA, and the sampling cone voltages were at 12, 20, 30, and 40 V. The acquisition at these four different cone voltages was carried out in one run using different channels. Data were acquired in full scan positive ion mode (*m/z* 50–600) and selected ion recording (SIR) at 10 Hz scan speed. Instrument control and MS data analysis were carried out using Mass Lynx *v*4.2 software (Waters Corporation, Manchester, UK).

### Sample analysis

Samples were commercial oily injection preparation vials from in-house stock and the presence of steroid esters had been checked previously by UHPLC–MS/MS. In total, seven oily injection preparations that could be used for illicit practices in cattle were analyzed. Before analysis, each oily injection preparation was diluted 100-fold in acetonitrile.

Sample introduction was carried out by spotting the diluted liquid extracts (2 μL) on a pre-cleaned glass capillary, immediately followed by insertion in the ionization source region.

### Quality control and method performance

Quality control standard solutions and procedural blanks were introduced among the analysis of samples to ensure the quality of the results and control the sensitivity of the ASAP–MS system. An oily injection preparation, free of target compounds, was used to prepare spiked samples at concentrations ranging 10–500 mg L^−1^ before the extraction procedure.

The matrix effect (ME, %) in the ionization process was estimated for each compound from the relative difference between the peak area observed in the analysis of the spiked blank oil diluted in acetonitrile and that obtained from standard mixtures prepared in acetonitrile:water (50:50, *v/v*) at the same concentration level. Method limits of detection (MLODs) were estimated as peak to peak S/N in matrix-matched solutions. Moreover, linearity within the working concentrations range (10–500 mg L^−1^) for both standard solutions and matrix-matched calibration solutions before dilution was studied. Intra-day precision as relative standard deviation (RSD, %) was estimated using spiked blank oily sample at concentrations ranging from 50 to 100 mg L^−1^.

### Data analysis

LiveID 2.0 software (Waters Corporation, Manchester, UK) was employed in library matching mode to identify compounds in a sample. A homemade library with seventeen steroids (Fig. S1) was created using an MSP mass spectral data storage format by MSP librarian software [23]. The LiveID software loads an MSP file extension in NIST Standard Reference Database format for library matching. Afterwards, LiveID reads MassLynx.raw data files, matches the compounds of the library file against each sample, and reports a score indicating the strength of the match for each compound. The LiveID software allows the user to carry out the library matching in both real-time and offline modes providing match scores in less than a minute. A library for steroid esters was created by recording mass spectra of each compound in one run at four different cone voltages (12, 20, 30, 40 V). To evaluate the performance of the developed ASAP–MS method and establish the library matching identification criteria, several mixtures of target compounds in both acetonitrile:water (50:50, *v/v*) solvent mixture and oily based blank matrix diluted in acetonitrile were prepared at 100 mg L^−1^ and analyzed following the workflow described in “Instrumentation” section. The match criteria using the library matching approach were set with a “hit” requiring a score ≥ 850 for channel 1 (cone voltage 12 V), score of ≥ 825 in the three remaining channels, and an average score of ≥ 800.

## Results and discussion

### ASAP–MS optimization

**Fig. 1:**
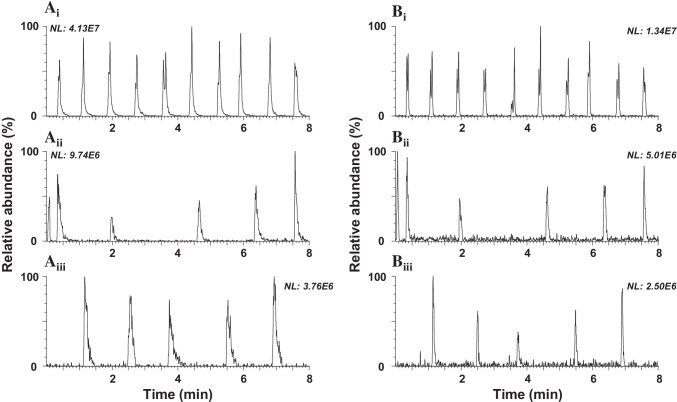
SIR chronograms obtained by ASAP–MS for **A** testosterone decanoate (*m/z* 443) and **B** estradiol dipropionate (*m/z* 385) using as sample introduction (i) pipetting 2 μL; (ii) dipping and analyzing directly; (iii) dipping and analyzing after drying

Sample introduction in an ambient ionization source should be performed in a consistent manner to obtain reproducible results. The obtained peak width using the ASAP-MS system is typically around 2 s. Since the MS consists of a quadrupole capable of scan rates, up to 20 Hz peaks can be reliably described with enough data points over the peak. In the ASAP–MS system, the sample is introduced by a disposable glass capillary. The sample is transferred by pipetting a known amount on the capillary or dipping the capillary into the sample. The obtained results by (i) pipetting 2 μL of a mixture of target compounds at 1 mg L^−1^ were compared with those obtained by dipping the capillary in an injection vial containing 0.4 mL solution of the target compounds at the same concentration and (ii) analyzing it directly or (iii) after waiting until complete solvent evaporation by leaving it in air conditions for 45 s. Wet conditions containing protic solvents (i and ii) in ASAP–MS usually lead to the formation of the protonated molecule [M + H]^+^ via proton transfer from the solvent components. In contrast, relatively dry ambient conditions (iii) can result in molecular ions [M]^+•^ via charge transfer from radical nitrogen [24]. However, in this case, since an open ASAP source has been employed, all the studied compounds showed [M + H]^+^ ions in their full scan mass spectra (Table S1), and under no circumstance was the molecular ion observed, in accordance with other studies [20]. These results indicate that ambient humidity may also play an important role in the protonation of these analytes when dried samples or non-protic solvents are used, as in other AIMS techniques such as DART [25]. As shown in Fig. 1 for (A) testosterone decanoate and (B) estradiol dipropionate, the signal response of target compounds is greater when introducing the extract by pipetting 2 μL than by dipping the capillary. When using the dipping approach, the lower signal is probably because only the tip of the glass capillary (~ 4 mm) was put below the solution’s surface using a vertical alignment which may produce the dropping of the sample and lower the sample volume. Also, dipping is less defined and will cause a higher variation in signal than that obtained when pipetting a fixed amount of the sample. The dry effect shows a two times lower signal response than without drying (Fig. 1). The precision differs between the three approaches showing RSD% values lower than 15% when pipetting and up to 50% when dipping. Dipping was found to be less reproducible than pipetting.

The scan time was also optimized; measurements at 2, 10, and 20 Hz were carried out. The precision was below 15% in all cases, but the signal response at 20 Hz was significantly lower, and the chronogram peaks of the target compounds were not well defined (Fig. S2). In general, at least 8 points are needed to describe a peak shape. Although the obtained results with a scan time of 2 and 10 Hz showed similar intensities, the chronogram was best described using 10 Hz, providing 12 data points per peak instead of 3 data points per peak in the case of 2 Hz, which are not enough data points to describe a peak.

The solvent and modifiers in which the compounds are dissolved can influence ASAP ionization efficiency. First, acetonitrile and methanol as organic solvents were evaluated in a mixture with water 1:1 (*v/v*). As expected, no differences between these two solvents were observed since water was present in both solvents. Second, the addition of a small percentage (0.1%) of modifiers with different gas-phase properties was evaluated. Different modifiers suitable for plasma-based ionization mechanisms such as toluene, acetone, formic acid, and acetic acid were added to acetonitrile solvent mixture with water 1:1 (*v/v*) to evaluate the ionization efficiency of the target compounds. None of these modifiers showed any significant improvement in the signal response of the target compounds (Fig. S3). For all further experiments, acetonitrile:water (50:50, *v/v*) was employed without additives.

**Fig. 2:**
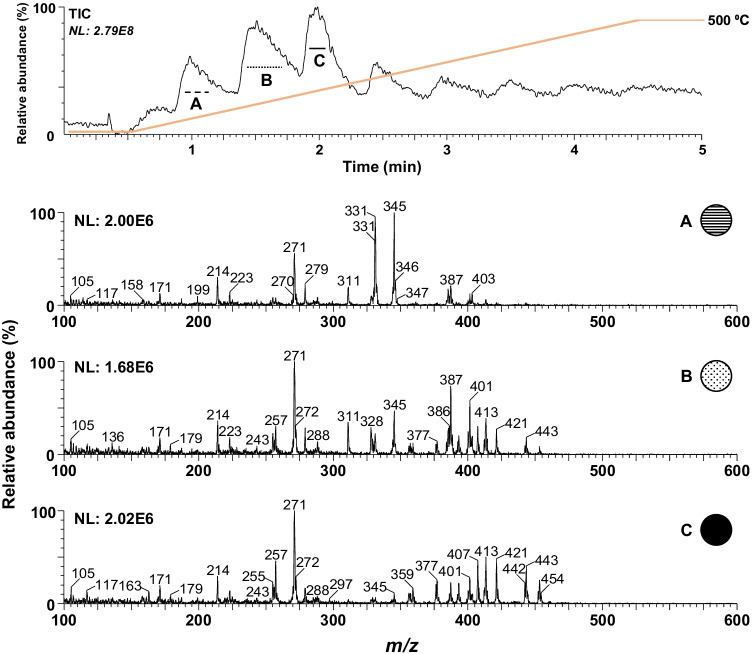
Upper trace, desorption temperature profile of the studied steroid esters; lower traces mass spectra of regions **A** (0.85–1.15 min), **B** (1.4–1.7 min), and **C** (1.9–2.1 min)

The probe temperature was optimized to obtain the compound’s complete vaporization without thermal degradation, which is essential for higher signal intensity. For this purpose, a mixture containing seventeen steroid ester standards was submitted to a temperature ramp from 100 to 500 °C with a 50 °C increase every 0.5 min. The mass range from *m/z* 100 to 600 was chosen to ensure that neither potential in-source fragmentation nor adduct ions formation of the analytes would be missed. As shown in Fig. 2, the ASAP–MS TIC trace exhibits several peaks referring to the vaporization of different compounds due to the temperature increase over time. The esters with short side chain such as T Ac (*m/z* 331) and T Pr (*m/z* 345) showed the lowest desorption temperature (200 °C), while as the temperature rises, the desorptions of those exhibiting a long side chain such as T En (*m/z* 401), T Cy (*m/z* 413), and T Dc (*m/z* 443) were observed. Since the side chain of target compounds showed to influence the probe temperature, the temperature ramp would provide additional identification related to the boiling point of the target compounds. However, it should be mentioned that several ions such as *m/z* 345 and *m/z* 401, which correspond to T Pr and T En, respectively, can be observed at different temperature regions (A–C), indicating that these temperatures are not the most efficient for complete desorption. More precise control of the temperature could regulate the exact moment of desorption from the probe. Currently, only steps of at least 50 °C are possible in the instrument. Therefore, to ensure the complete desorption of all target compounds simultaneously, the probe temperature was set at 450 °C, which is high enough to efficiently desorb all steroid esters from the glass probe.

### Library creation and identification criteria

A single quadrupole mass analyzer, working at low mass resolution, with ambient ionization techniques such as ASAP that does not include chromatographic separation of the analytes can hamper the unequivocal identification of compounds and suffer from insufficient specificity when operated in full scan mode. The use of in-source fragmentation has been shown to improve the specificity of the technique when used in the forensic field [26, 27].

**Fig. 3:**
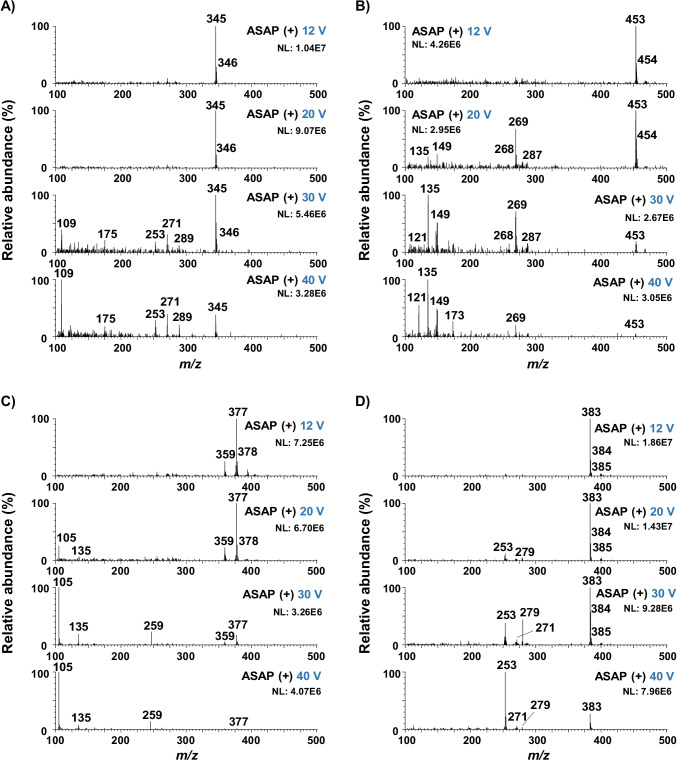
Mass spectra for **A** testosterone propionate, **B** boldenone undecylenate, **C** estradiol benzoate, and **D** trenbolone enanthate standards by ASAP–MS at the four selected cone voltages: 12, 20, 30, and 40 V

Initially, 15, 30, 50, and 70 V were applied on the selected steroid esters in this study. However, the two highest voltages showed excessive fragmentation producing poor spectra for library matching, and therefore, the use of these voltages did not provide enough information about each target compound. The behavior of the target compounds at lower voltages was further studied at cone voltages of 12, 20, 30, and 40 V. These voltages allowed the characterization of the steroid esters and provided structural information of the target compounds. The mass spectra obtained with the selected four cone voltage values for (A) testosterone propionate, (B) boldenone undecylenate, (C) estradiol benzoate, and (D) trenbolone enanthate are depicted in Fig. 3. As can be observed, in all cases, the lowest cone voltage showed the [M + H]^+^ as the base peak of the mass spectrum, while as the voltage value increases, there is a greater number of characteristic fragments in the mass spectra of each compound (Table S1). The observed fragment ions agree with those reported by Doue et al. [20] using an ASAP–MS/MS system. The assignment and structure of these fragment ions have been proposed in the literature [28–30]. In the case of testosterone-based compounds (Fig. 3A), *m/z* 271 [C_19_H_27_O]^+^ which is present in all testosterone esters corresponds to the ester cleavage of the [M + H]^+^ molecule. Additionally, other common ions such as *m/z* 109 [C_7_H_9_O]^+^ and *m/z* 105 [C_7_H_5_O]^+^ which are observed by ASAP–MS for this family of compounds have been assigned as B-ring cleavage products [30, 31]. In the case of boldenone undecylenate, fragment ions of boldenone such as *m/z* 269 [C_19_H_25_O]^+^ correspond to the ester cleavage of the [M + H]^+^ molecule but also *m/z* 135 [C_10_H_15_]^+^ and *m/z* 121 [C_8_H_9_O]^+^ (Fig. 3B) corresponding to the fission of B-ring [32] were observed. The *m/z* 135 fragment ion was also observed in the fragmentation studies of estradiol ester compounds (Fig. 3C). Moreover, the estradiol esters E2 V1 and E2 DiPr showed fragment ions at *m/z* 255 [C_18_H_23_O]^+^ that yield from the loss of R-COOH [30], and *m/z* 105 [C_7_H_5_O]^+^ corresponding to a phenylcarbonyl group [33]. Trenbolone esters showed a fragment ion *m/z* 271 [C_18_H_23_O_2_]^+^, which correspond to the ester cleavage of the [M + H]^+^ molecule and a consecutive fragment ion due to the loss of a water molecule from the *m/z* 271 to yield the fragment ion at *m/z* 253 [C_18_H_21_O]^+^ [29] (Fig. 3D). In Fig. 3D is also a fragment ion *m/z* 279 present, probably due to ring cleavage, but the pathway is unclear.

To build a library of the selected steroid esters with the provided library tool, MSP librarian, a method combining four acquisitions in full scan mode with different cone voltages to promote in-source fragmentation was used. For this purpose, first full scan mass spectra of the steroid esters at each cone voltage were recorded. Second, all *m/z* peaks present in the mass spectra above 5% threshold (no background subtraction was applied) were added to a library. To classify samples against the build library, the scores of each acquired spectrum at each cone voltage are combined as weight average, and the same threshold to select relevant *m/z* peaks of 5% as during the library creation was used. Samples are classified not only at each independent cone voltage but also as an average for all four cone voltages.

First, the performance of the library matching identification was studied with the standard mixtures. They generally showed high match scores, resulting in average and independent scores for the different cone voltages above 900 and 850 (Table S2). When the target compounds are analyzed individually (Table S2, solutions 1–4), their match score is higher than when they are analyzed in mixtures with other compounds (solutions 5–8, Table S2). This is due to the fact that the obtained mass spectra are the sum of all the generated ions for the 17 steroid esters since there is no chromatographic separation to extract individual mass spectra for each target compound. Therefore, the number of ions present in the mass spectra at each cone voltage results in more *m/z* ions than those recorded in the library for the individual compounds, reducing the match score. Some false-positive results were generated using the library approach for identification. In those cases, although the average match score was high (> 800), the match score values at individual cone voltage settings were lower than 800. For instance, when E2 DiPr was present in the solution, the library also identified E2 V1. Both compounds are not correctly classified due to the structural similarity of the spectra of these compounds at high voltage values (30–40 V). These two esters are both based on estradiol. They both present common fragment ions such as *m/z* 279, *m/z* 255, *m/z* 159, and *m/z* 109 in their mass spectra leading to similar library matching results (Table S1).

Second, to evaluate the library performance in samples, blank oily solutions were spiked. The recorded spectra of these samples are more complex due to the presence of the matrix components. The presence in the mass spectra of ions derived from the matrix results in an overall decrease in the match scores, although they remain high (> 800) for the spiked samples (Table S3). Nevertheless, it leads to a higher number of compounds wrongly identified by the library matching due to interfering ions from the matrix resulting up to 28% of false-positive and 10% false-negative identification. This is a recognized drawback of employing in-source fragmentation over precursor ion selection if a triple quadrupole is used. Moreover, the match score results obtained in the analysis of these compounds both in individual solutions and in a mixture are not equal and thus, these differences must also be taken into account when establishing the acceptable match criteria when identifying a compound in a sample. All spiked analytes gave a positive identification above for the average score of > 800 (Table S3), which is slightly lower than in pure standards (> 850). When the scores at the different channel cone voltages were examined, it was noted that at a cone voltage of 12 V, a score of > 850 was obtained, and for the other three cone voltages, a score of > 825. From this experiment, it was determined that the optimal match criteria for samples are as follows: average score of ≥ 800, channel 1 (cone voltage 12 V) score ≥ 850, and a score of ≥ 825 in the three remaining channels in order to reduce the false-positive identifications to 7%.

### ASAP–MS performance in samples

For the analysis of steroid esters in oily preparations by ASAP–MS/MS, Doue et al. proposed a quick, cost-effective, and straightforward 1,000-fold dilution with methanol before analysis since the concentration of steroid esters is very high in these types of samples [20]. It must be taken into account that their method used selected reaction monitoring (SRM) acquisition mode, which has greater sensitivity and selectivity than a full scan acquisition mode on a single quadrupole instrument. Therefore, in this study, both the dilution range and the type of solvent for dilution were evaluated by spiking a blank oil solution with 10 mg L^−1^ of the target compounds and diluting with acetonitrile, toluene, and methanol as organic solvents and recording the full scan mass spectra. To analyze oil samples, water was avoided since this may result in an inhomogeneous solubility of the samples. At the same time, as mentioned above, these differences in the solvent composition between standards and samples did not produce any change in the mass spectra since only the protonated molecule was obtained in all cases. The 1,000-fold dilution results did not permit detection of any of the steroid esters in the sample, whereas most target compounds could be identified when carrying out a 100-fold dilution. These initial experiments using different organic solvents showed that the type of organic solvent greatly affects the measured mass spectra of steroid esters in the oil samples. The difference in spectra can be explained by the solubility of the steroid esters vs. oil matrix compounds in the different organic solvents. Among the selected organic solvents, the highest-quality and informative mass spectra, in terms of both signal intensity and target compounds extraction, were provided by acetonitrile (Fig. S4A). The obtained spectra using toluene as organic solvent (Fig. S4B) showed a greater number of ions at the lowest range of the mass spectra (*m/z* 100–300). At the same time, methanol extracted compounds generated ions with higher *m/z* values (Fig. S4C), leading to possible future interferences with the selected target compounds. For this reason, acetonitrile was chosen as the dilution solvent for samples.

One of the main concerns in the ambient ionization techniques is the carry-over since it can provide a high risk of cross contaminations yielding high numbers of false-positive screening results. The analysis of diluted extracts already reduces the risk of carry-over and/or contamination. To ensure no carry-over occured, the glass probe was spiked with steroid esters once, and 6 consecutive analyses without cleaning the rods in between. No carry-over effect or source contamination was observed as previously reported for the use of ASAP–MS in other studies [33].

Additionally, the components in the sample can influence the analyte’s response by suppressing or enhancing ionization and thereby affect the method’s reproducibility and accuracy. Therefore, the effect of the matrix on the response was evaluated using both calibration curves obtained from the analysis of standards prepared in acetonitrile:water (50:50, *v/v*) and matrix-matched standards in acetonitrile (Fig. S5). Results showed that the matrix affects the intensity for most analytes (Table 1). Thus, if quantitation of steroid esters in samples would be desirable with the ASAP–MS system, correction for signal loss should be employed, such as the use of internal standards or matrix-matched calibration curves.

**Table 1:** Performance parameters of the ASAP–MS method estimated in oil samples

Compound	MLODs (mg L^−1^)	Matrix effect (%)		Intra-day precision (RSD, %)	
		30 mg L^−1^	100 mg L^−1^	30 mg L^−1^	100 mg L^−1^
T Ac	2	33	− 3	69	7
T Pr	4	− 71	− 24	31	16
T Iso	4	1	− 49	49	17
T En	5	− 13	− 31	19	23
T Dc	6	− 28	− 41	22	21
T Bz	6	− 35	− 35	18	18
T PhPr	8	− 11	− 33	33	6
T Cy	2	8	13	7	24
N PhPr	8	− 32	− 41	20	18
B Un	18	− 23	− 28	31	7
E2 DiPr	5	0	5	19	23
E2 V1	20	− 30	− 21	36	13
E2 Bz	22	− 14	− 11	30	4
Tr	1	− 23	− 20	8	26
Tr Ac	2	168	105	22	19
Tr En	2	51	− 9	31	2
D En	4	9	− 15	30	15

Nevertheless, the linearity of the analytical response of target compounds within the working range of 10–500 mg L^−1^ was satisfactory obtaining correlation coefficients (*r*) higher than 0.977 for all the compounds. MLODs were estimated and these values ranged from 1 to 8 mg L^−1^ except for B Un, E2 V1, and E2 Bz, which were 18, 20, and 22 mg L^−1^, respectively (Table 1). The intra-day precision was determined using the corresponding blank sample spiked at two concentration levels (described in “Quality control and method performance” section) and results showed RSD% values between 2 and 30% at 30 mg L^−1^ for most of the compounds whereas at 100 mg L^−1^, RSD% values were lower than 25%.

### Analysis and identification of steroids in injection preparations

As a response to the potential illicit application of steroids in doping or in food production systems, the applicability of the developed ASAP–MS workflow was evaluated by analyzing seven injection preparations that were previously characterized by LC–MS/MS analysis. The samples were prepared and analyzed as described in “Sample analysis” section and the corresponding screening matches were based on a single analysis pipetting 2 μL onto the glass capillary probe. A notable presence of detector saturation was observed when analyzing oily samples. Saturation means that the intensity for one or more *m/z* values within the scan reached the maximum value for the instrument. When one or more *m/z* peaks are saturated, the relative intensity values of different peaks within the scan may not be accurately represented, and thus, the data quality for quantitation is compromised. Therefore, this study focused on the identification of steroid esters in oil preparations only.

**Fig. 4:**
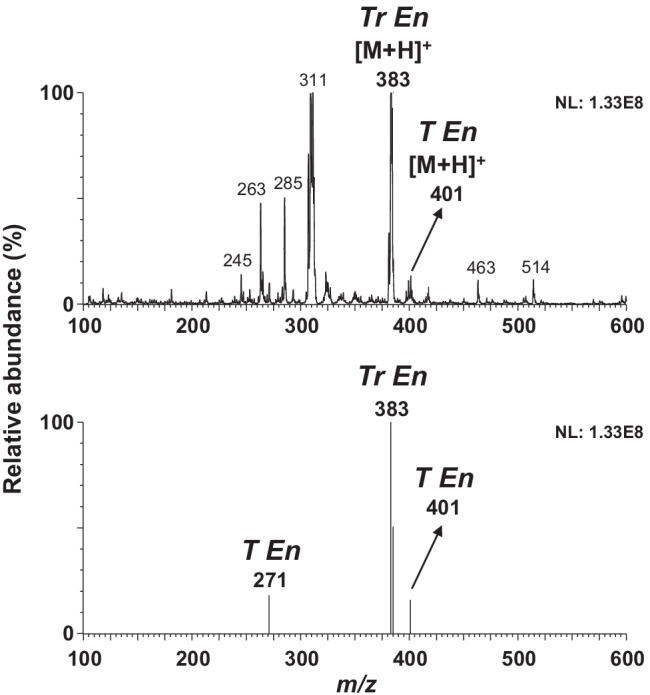
ASAP–MS full scan and selected ion recording spectra of sample N° 4. Selected *m/z* values acquired were as follows: T Ac, 331; T Pr, 345; T Iso, 289; T En, 401; T Dc, 443; T Bz, 393; T PhPr, 421; T Cy, 413; N PhPr, 407; B Un, 453; E2 DiPr, 385; E2 V1, 357; E2 Bz, 377; Tr, 271; Tr Ac, 313; Tr En, 383; D En, 417

In this case, the database searching approach was carried out automatically by the LiveID software tool (Fig. S6) showing an advantage over the previous method developed by McCullough et al. [27] using an ASAP-QDa benchtop system for the analysis of drug seizures. Table 2 summarizes the library matching identification results for the seven oily preparations analyzed and are compared with confirmatory LC–MS/MS. As can be observed from Table 2, the major constituent identified by the ASAP–MS library matching agrees with the LC–MS/MS analysis. The missed identification of compounds was related to the higher MLODs observed with the ASAP–MS technique than the LC–MS/MS measurement, which can detect trace amounts. The missed identification for nandrolone decanoate in samples N° 3 and N° 6 was attributed to the fact that this compound was not included in the list of steroid esters selected for the library creation. Therefore, it was not in the database, so it could not correctly be identified in these samples. Nevertheless, the *m/z* 275 probably corresponding to the [M + H]^+^ of nandrolone could be observed in the full scan mass spectra at 12 and 20 cone voltages. Moreover, at higher cone voltage values (30–40 V), *m/z* ions previously proposed as nandrolone decanoate product ions such as 257, 155, and 109 [34, 35] could be observed pinpointing its presence in the sample. Trenbolone-based compounds such as Tr, Tr En, and Tr Ac showed high average match scores (≥ 800) in many cases (samples N° 1–5). However, their match scores in channels 2–4 were below 800 and therefore, they were not considered as hits in these cases. These results are a consequence of the complexity of these samples containing cocktails of steroid esters and the degree of fragmentation similarity between steroid esters. It should be noted that in “Library creation and identification criteria” section, spiked samples with testosterone isocaproate both were identified when spiked individually and in the presence of other steroid esters at 100 mg L^−1^. In contrast, in these real samples, the library matching approach was not able to identify testosterone isocaproate. This might be due to the presence of interfering ions from the other steroid esters and/or matrix in the mass spectra. Still, the identification of other steroid esters in these samples would flag these samples as suspicious, and a follow-up conducting a targeted LC–MS/MS method for steroid esters would be employed. Figure 4A shows an example of the full scan mass spectra (cone voltage 12 V) of sample N° 4. Several peaks potentially related to the [M + H]^+^ of the studied steroid esters can be identified from this spectrum. For instance, *m/z* 383 and *m/z* 401 correspond to trenbolone enanthate and testosterone enanthate, respectively. Library searching of this data results in hits for trenbolone enanthate, testosterone enanthate, drostanolone enanthate, and trenbolone. Nevertheless, library matching results in the presence of drostanolone enanthate in this sample, whereas boldenone undecylenate was identified by the LC–MS/MS method. It may be possible to reduce the incidence of these incorrect identifications by using stricter identification criteria during library matching, but this would increase the number of false-negative samples. A screening technique like this aims to filter out suspicious samples for follow-up analysis, and a higher number of false-positive results is acceptable.

**Table 2:**
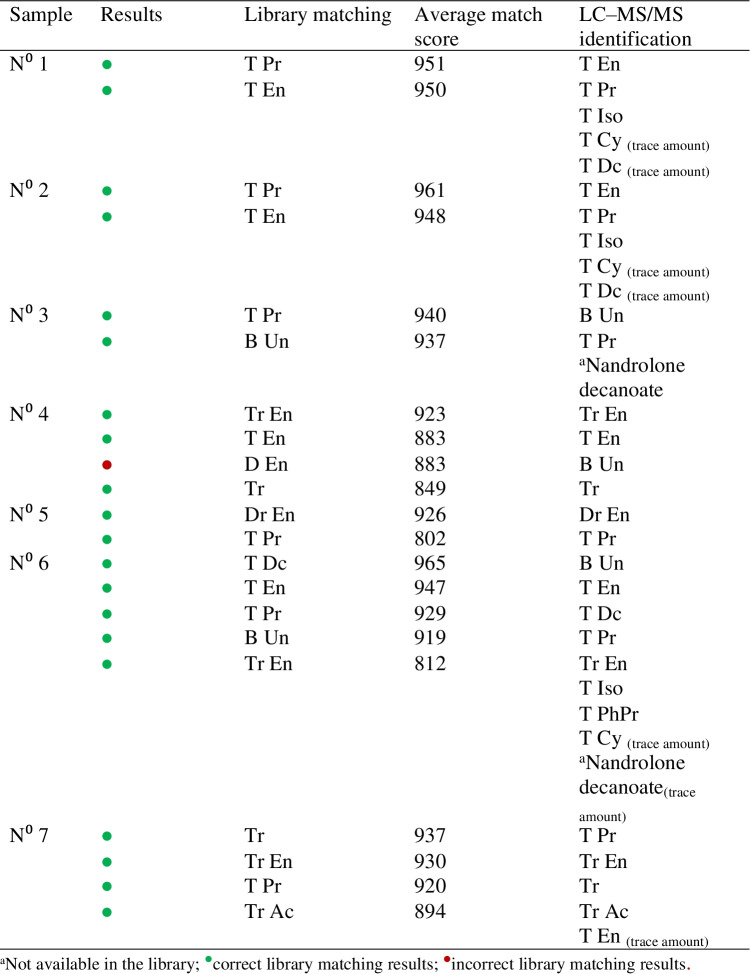
Screening results by ASAP–MS LiveID library matching compared with LC–MS/MS

In addition to the full scan acquisition mode, selected ion recording acquisition can be employed, which could be used to support the obtained results from the library matching. For this reason, all samples were also analyzed by a SIR mode method which includes the [M + H]^+^ ion of the seventeen steroid esters at the lowest cone voltage value (12 V). Figure 4B shows the SIR mass spectra of sample N° 4. As can be observed, the presences of trenbolone (*m/z* 271), trenbolone enanthate (*m/z* 383), and testosterone enhantate (*m/z* 401) are in agreement with the library matching results. Nevertheless, the *m/z* 417 corresponding to the hit of drostanolone enanthate was not observed, being in agreement with the LC–MS/MS results, indicating a false-positive identification with the library matching approach. Thereby, the additional use of SIR acquisition mode, which leads to results within 2 min, would improve the screening results of the library matching.

## Conclusions

The potential of ASAP single quadrupole MS system has been evaluated for the rapid screening of anabolic steroid esters. The library match tool is used to identify the spectral data acquired at four stages of in-source fragmentation (12, 20, 30, and 40 V) to score the match between the library and the analyzed sample. Such a simple analytical workflow enables analysis of a large number of samples in a short analysis time (1 min) using a small sample (2 μL) in a fully automated data interpretation process. The samples can be identified based on the library matching by in-source fragmentation patterns observed and selected ion recording acquisition mode. The applicability was demonstrated by identifying anabolic steroid esters such as testosterone, trenbolone, and estradiol in real samples. This approach can be beneficial to support the control and enforcement authorities since it can rapidly pinpoint suspicious samples for further confirmatory analysis. Food safety, anti-doping, and forensic laboratories are looking for high-throughput screening methods for counterfeited substances, and these initial results demonstrate the applicability of the simplified ASAP–MS method. Furthermore, since the mass spectrometer can be operational in an hour, it has the potential to be used for future on-site analysis of organic compounds in liquid samples.

## Supplementary Information

Below is the link to the electronic supplementary material.

Supplementary file1 (PDF 626 KB)
