# Supplementary material for: Simplified screening approach of anabolic steroid esters using a compact atmospheric solid analysis probe mass spectrometric system
Source: Anal Bioanal Chem. 2022 Feb 27;414(11):3459–70. doi: 10.1007/s00216-022-03967-y (PMC9018663; doi:10.1007/s00216-022-03967-y)
Supplement: Supplementary file 1 — Supplementary file1 (PDF 626 KB) [file 216_2022_3967_MOESM1_ESM.pdf]

Supplementary material

Simplified screening approach of anabolic steroid esters using a compact Atmospheric Solid Analysis Probe mass spectrometric system

Ane. Arrizabalaga-Larrañaga[(1)]*, Paul W. Zoontjes[(2)], Johan J. P. Lasaroms[(2)], Michel W. F. Nielen[(2,3)], Marco H. Blokland[(2)]

[(1)] Department of Chemical Engineering and Analytical Chemistry, University of Barcelona. Av. Diagonal 645, E-08028, Barcelona, Spain

[(2)] Wageningen Food Safety Research (WFSR), Part of Wageningen University & Research, P.O. Box 230, 6700 AE Wageningen, The Netherlands

[(3)] Wageningen University, Laboratory of Organic Chemistry, Stippeneng 4, 6708 WE Wageningen, The Netherlands

* Corresponding author: Ane Arrizabalaga-Larrañaga
Email: a.arrizabalaga@ub.edu

Table of Contents

Supplementary Tables 2

Table S1: ASAP–MS in-source fragmentation tentative assignments for studied steroids. 2

Table S2: Results of the library matching of the home-made standard mixtures based on the average and individual stage match scores. 5

Table S3: Results of the library matching of the spiked blank oils (100 mg L^{-1}) based on the average and individual stage match scores. 6

Supplementary Figures 7

Fig. S1: Chemical structures and elemental composition of the studied steroid esters..... 7

Fig. S2: Chronograms obtained by ASAP−MS for (A) Testosterone propionate and (B) nortestosterone phenylpropionate using scan time 2, 10, and 20 Hz........ 8

Fig. S3: Effect of the ASAP–MS solvent additive in the ASAP-MS signal for some representative esteroid esters. 8

Fig. S4: Full Scan mass spectra of a spiked blank sample with target compounds at 100 mg L^{-1} using (A) acetonitrile, (B) toluene, and (C) methanol. 9

Fig. S5: External standard calibration (blue) and matrix matched calibration (orange) curves of (A) Tr Pr, (B) E2 DiPr, (C) T Dc, and (D) Tr in the working range (10-500 mg L^{-1}). 10

Fig. S6: LiveID software interface workflow for non-expert users to apply the method to real samples. 11

Supplementary Tables

Table S1: ASAP–MS in-source fragmentation tentative assignments for studied steroids.

Compound	Cone (V)	*m/z*	Ion Assignment
T Ac	12	331	$[M+H]^+$
	30	331	$[M+H]^+$
		289	$[C_{19}H_{29}O_2]^+$
		271	$[C_{19}H_{27}O]^+$
		247	$[C_{17}H_{27}O]^+$
		218	$[C_{15}H_{22}O]^{+\bullet}$
		187	$[C_{13}H_{15}O]^+$
		107	$[C_7H_7O]^+$
T Pr	12	345	$[M+H]^+$
	30	345	$[M+H]^+$
		271	$[C_{19}H_{27}O]^+$
		253	$[C_{19}H_{25}]^+$
		175	$[C_{12}H_{15}O]^+$
		109	$[C_7H_9O]^+$
T Iso	12	387	$[M+H]^+$
	30	387	$[M+H]^+$
		289	$[C_{19}H_{29}O_2]^+$
		271	$[C_{19}H_{27}O]^+$
		175	$[C_{12}H_{15}O]^+$
		109	$[C_7H_9O]^+$
T En	12	401	$[M+H]^+$
	30	401	$[M+H]^+$
		271	$[C_{19}H_{27}O]^+$
		253	$[C_{19}H_{25}]^+$
		175	$[C_{12}H_{15}O]^+$
		113	$[C_7H_{13}O]^+$
		109	$[C_7H_9O]^+$
T Dc	12	443	$[M+H]^+$
	30	443	$[M+H]^+$
		271	$[C_{19}H_{27}O]^+$
		211	$[C_{13}H_{23}O_2]^+$
		155	$[C_9H_{15}O_2]^+$
		121	$[C_8H_9O]^+$
		109	$[C_7H_9O]^+$
T Bz	12	393	$[M+H]^+$
	30	393	$[M+H]^+$
		271	$[C_{19}H_{27}O]^+$
		253	$[C_{19}H_{25}]^+$
		175	$[C_{12}H_{15}O]^+$
		109	$[C_7H_9O]^+$
		105	$[C_7H_5O]^+$

Table S1 (cont.) : ASAP–MS in-source fragmentation tentative assignments for studied steroids.

Compound	Cone (V)	*m/z*	Ion Assignment
T PhPr	12	421	$[M+H]^+$
	30	421	$[M+H]^+$
		271	$[C_{19}H_{27}O]^+$
		253	$[C_{19}H_{25}]^+$
		133	$[C_9H_9O]^+$
		105	$[C_8H_9]^+$
T Cy	12	413	$[M+H]^+$
	30	413	$[M+H]^+$
		271	$[C_{19}H_{27}O]^+$
		253	$[C_{19}H_{25}]^+$
		125	$[C_8H_{13}O]^+$
		109	$[C_7H_9O]^+$
		107	$[C_7H_7O]^+$
N PhPr	12	407	$[M+H]^+$
	30	407	$[M+H]^+$
		275	$[C_{18}H_{27}O_2]^+$
		257	$[C_{18}H_{25}O]^+$
		239	$[C_{18}H_{23}]^+$
		133	$[C_9H_9O]^+$
		105	$[C_8H_9]^+$
B Un	12	453	$[M+H]^+$
	30	453	$[M+H]^+$
		287	$[C_{19}H_{27}O_2]^+$
		269	$[C_{19}H_{25}O]^+$
		268	$[C_{19}H_{24}O]^{+\bullet}$
		149	$[C_{11}H_{17}]^+$
		135	$[C_{10}H_{15}]^+$
		121	$[C_8H_9O]^+$
E2 DiPr	12	385	$[M+H]^+$
		311	$[C_{21}H_{27}O_2]^+$
	30	385	$[M+H]^+$
		329	$[C_{21}H_{29}O_3]^+$
		311	$[C_{21}H_{27}O_2]^+$
		279	$[C_{20}H_{23}O]^+$
		255	$[C_{18}H_{23}O]^+$
		159	$[C_{11}H_{11}O]^+$
		135	$[C_8H_7O_2]^+$
		109	$[C_7H_9O]^+$

Table S1 (cont.) : ASAP–MS in-source fragmentation tentative assignments for studied steroids.

Compound	Cone (V)	*m/z*	Ion Assignment
E2 V1	12	357	$[M+H]^+$
		255	$[C_{18}H_{23}O]^+$
	30	357	$[M+H]^+$
		279	$[C_{20}H_{23}O]^+$
		255	$[C_{18}H_{23}O]^+$
		159	$[C_{11}H_{11}O]^+$
		133	$[C_{10}H_{13}]^+$
		109	$[C_8H_{13}]^+$
		105	$[C_8H_9]^+$
E2 Bz	12	377	$[M+H]^+$
	30	377	$[M+H]^+$
		259	$[C_{18}H_{27}O]^+$
		135	$[C_{10}H_{15}]^+$
		105	$[C_7H_5O]^+$
Tr	12	271	$[M+H]^+$
	30	271	$[M+H]^+$
		253	$[C_{18}H_{21}O]^+$
		199	$[C_{14}H_{15}O]^+$
		157	$[C_{12}H_{13}]^+$
		139	$[C_9H_{15}O]^+$
		129	$[C_8H_{17}O]^+$
		107	$[C_7H_7O]^+$
Tr Ac	12	313	$[M+H]^+$
	30	313	$[M+H]^+$
		279	$[C_{19}H_{35}O]^+$
		271	$[C_{18}H_{23}O_2]^+$
		253	$[C_{18}H_{21}O]^+$
Tr En	12	383	$[M+H]^+$
	30	383	$[M+H]^+$
		279	$[C_{19}H_{35}O]^+$
		271	$[C_{18}H_{23}O_2]^+$
		253	$[C_{18}H_{21}O]^+$
D En	12	417	$[M+H]^+$
		287	$[C_{20}H_{31}O]^+$
	30	417	$[M+H]^+$
		287	$[C_{20}H_{31}O]^+$
		279	$[C_{19}H_{35}O]^+$

Table S2: Results of the library matching of the home-made standard mixtures based on the average and individual stage match scores.

Sample	Compounds included	Compounds identified	Average match score	Cone voltage 12 score	Cone voltage 20 score	Cone voltage 30 score	Cone voltage 40 score
Solution 1	T Iso	T Iso	966	957	963	986	970
Solution 2	Tr Ac	Tr Ac	976	954	996	980	997
Solution 3	D En	D En	981	972	978	993	988
Solution 4	E2 DiPr	E2 DiPr	958	975	970	912	959
		E2 V1	902	867	913	924	938
Solution 5	T Pr	T Pr	976	979	977	982	964
	T Dc	T Dc	940	924	969	950	935
	N PhPr	N PhPr	932	902	935	943	981
	E2 DiPr	E2 DiPr	869	858	874	829	922
		E2 V1	856	860	856	**778**	825
Solution 6	T Pr	T Pr	972	979	979	972	951
	T Iso	T En	967	964	973	968	967
	T En	T Iso	930	885	973	972	934
Solution 7	T Pr	Tr En	970	981	938	972	980
	T Dc	T Pr	949	976	975	944	874
	N PhPr	N PhPr	948	939	947	942	976
	E2 Di	T Dc	937	951	959	962	860
	Tr En	E2 V1	850	860	846	**761**	922
		E2 Di	825	802	842	804	876
Solution 8	T Iso	T Iso	948	908	968	983	972
	B Un	B Un	942	942	916	972	939
	D En	E2 V1	930	937	900	905	968
	E2 V1	T Cy	929	887	947	935	987
	T Cy	D En	905	893	899	879	963

Table S3: Results of the library matching of the spiked blank oils (100 mg L^{-1}) based on the average and individual stage match scores.

Sample	Compounds included	Compounds identified	Average match score	Cone voltage 12 score	Cone voltage 20 score	Cone voltage 30 score	Cone voltage 40 score
Matrix 1	T Pr	N PhPr	982	992	949	995	985
	T Dc	T Pr	982	979	993	976	982
	N PhPr	T Dc	970	982	967	964	956
	E2 DiPr	E2 DiPr	879	***787***	953	911	958
		E2 V1	870	***798***	934	943	880
Matrix 2	T Ac	T Pr	981	977	988	973	988
	T Pr	T Ac	966	968	935	985	975
	B Un	B Un	889	945	868	897	911
Matrix 3	T PhPr	N PhPr	980	976	964	997	988
	T Cy	T PhPr	963	974	986	928	955
	N PhPr	T Cy	940	907	972	925	989
Matrix 4	T En	T En	892	916	905	931	790
Matrix 5	T Pr	Tr En	969	985	955	942	980
	Tr Ac	Tr Ac	934	925	1000	873	946
	N PhPr	T Pr	878	959	952	951	670
	E2 Di	N PhPr	844	903	902	833	679
Matrix 6	E2 DiPr	E2 V1	974	984	976	984	943
	E2 V1	E2 Di	831	859	835	822	1000
	E2 Bz	E2 Bz	909	851	989	853	975
Matrix 7	T Iso	E2 V1	877	925	896	810	825
	D En	D En	854	850	944	872	805
	E2 V1	T Iso	851	945	800	918	745
	T Cy						

Supplementary Figures

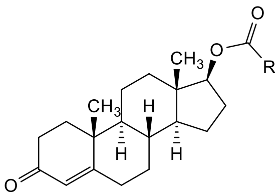

Testosterone esters (Tx)

Compound	Acronym	R	Chemical Formula
Testosterone acetate	T Ac	$-CH_3$	$C_{21}H_{30}O_3$
Testosterone propionate	T Pr	$-CH_2CH_3$	$C_{22}H_{32}O_3$
Testosterone isocaproate	T Iso	$-(CH2)_2CH(CH_3)CH_3$	$C_{25}H_{38}O_3$
Testosterone enanthate	T En	$-(CH_2)_5CH_3$	$C_{26}H_{40}O_3$
Testosterone decanoate	T Dc	$-(CH_2)_8CH_3$	$C_{29}H_{46}O_3$
Testosterone benzoate	T Bz	−Ph	$C_{26}H_{32}O_3$
Testosterone phenylpropionate	T PhPr	$-(CH_2)_2Ph$	$C_{28}H_{36}O_3$
Tetsosterone cypionate	T Cy	$-(CH_2)_2(C_5H_9)$	$C_{27}H_{40}O_3$

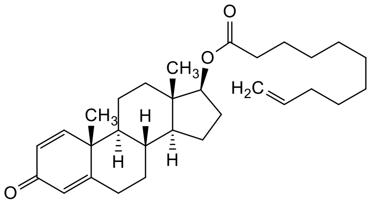

Boldenone undecylenate (B Un)
$C_{30}H_{33}O_3$

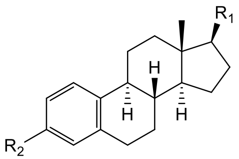

Estradiol esters (E2x)

Compound	Acronym	R_1	R_2	Chemical Formula
Estradiol dipropionate	E2 DiPr	$–C(O)CH_2CH_3$	$–OC(O)CH_2CH_3$	$C_{24}H_{32}O_4$
Estradiol valerate	E2 V1	–OH	$–OC(O)(CH_2)_3CH_3$	$C_{23}H_{32}O_3$
Estradiol benzoate	E2 Bz	–OH	–OC(O)Ph	$C_{25}H_{28}O_3$

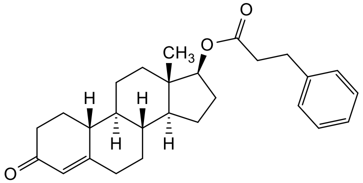

Nortestosterone phenylpropionate (N PhPr)
$C_{27}H_{34}O_3$

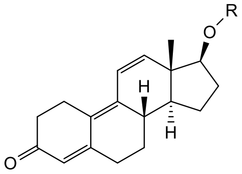

Trenbolone esters (Trx)

Compound	Acronym	R_1	Chemical Formula
Trenbolone	Tr	–H	$C_{20}H_{24}O_3$
Trenbolone acetate	Tr Ac	$–C(O)CH_3$	$C_{25}H_{34}O_3$
Trenbolone enanthate	Tr En	$–C(O)(CH_2)_5CH$	$C_{27}H_{44}O_3$

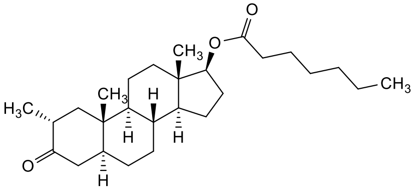

Drostanolone enanthate (D En)
$C_{27}H_{34}O_3$

Fig. S1: Chemical structures and elemental composition of the studied steroid esters.

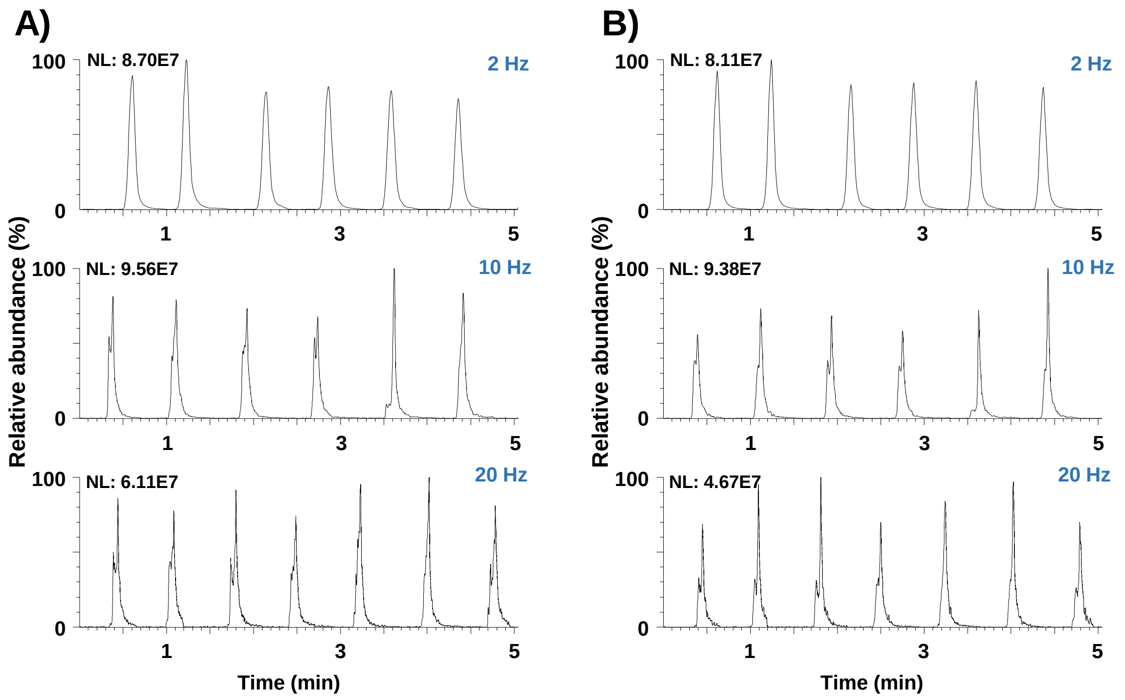

Fig. S2: Chronograms obtained by ASAP−MS for (A) Testosterone propionate and (B) nortestosterone phenylpropionate using scan time 2, 10, and 20 Hz.

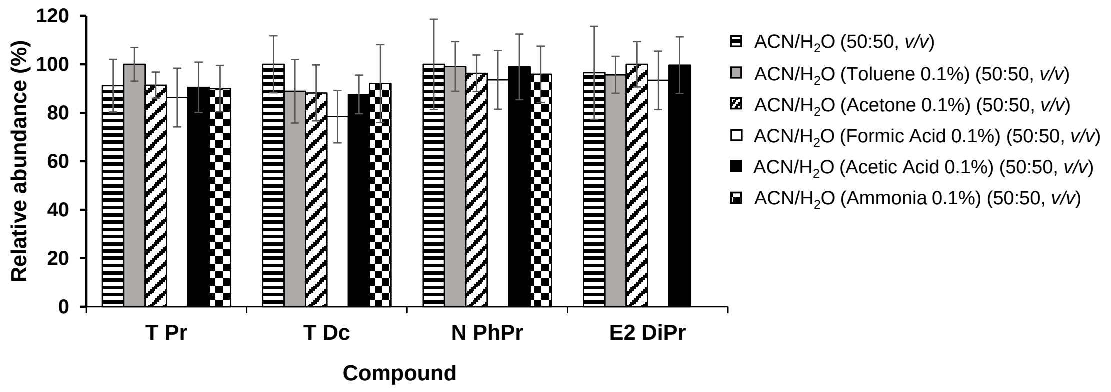

Fig. S3: Effect of the ASAP–MS solvent additive in the ASAP-MS signal for some representative esteroid esters.

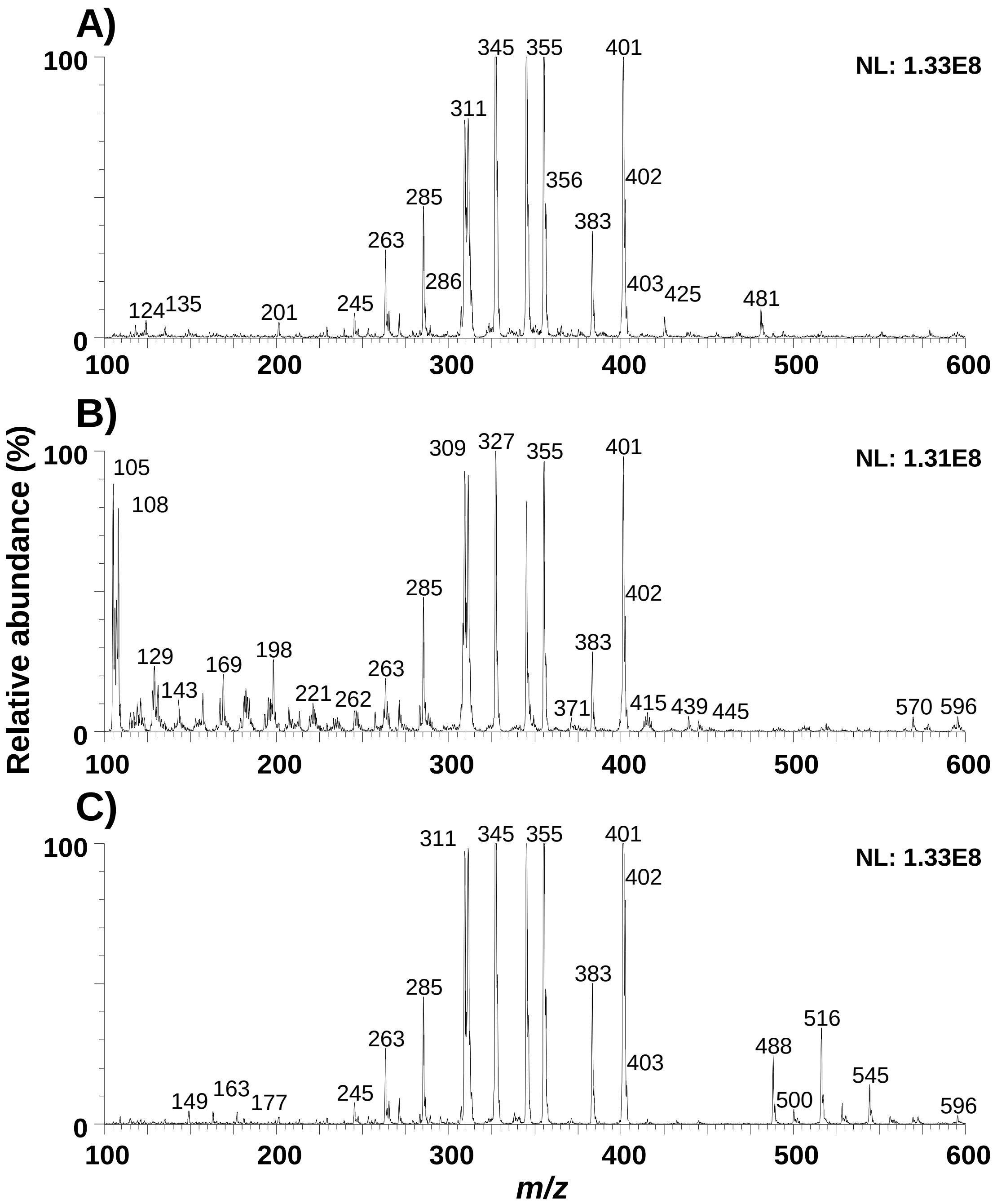

Fig. S4: Full Scan mass spectra of a spiked blank sample with target compounds at 100 mg L^{-1} using (A) acetonitrile, (B) toluene, and (C) methanol.

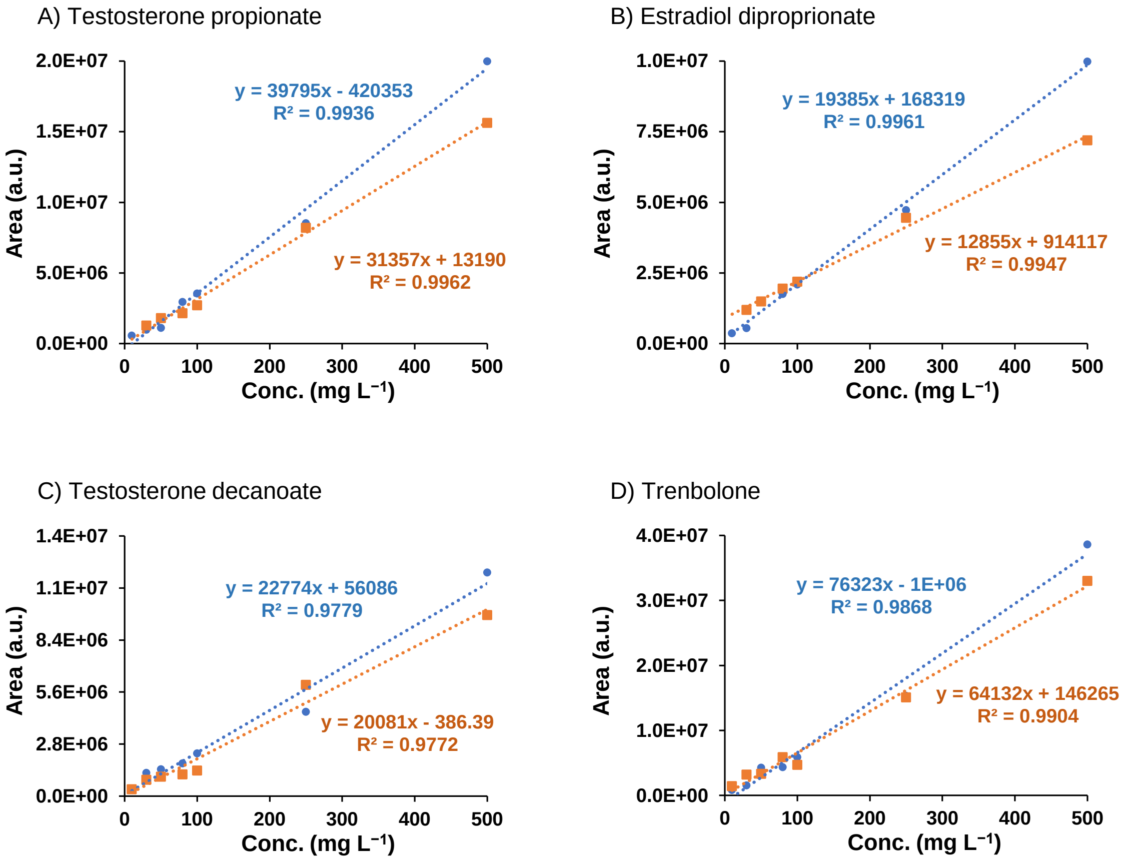

Fig. S5: External standard calibration (blue) and matrix matched calibration (orange) curves of (A) Tr Pr, (B) E2 DiPr, (C) T Dc, and (D) Tr in the working range (10-500 mg L^{-1}).

Stablished match criteria

Channel 1	Channel 2	Channel 3	Channel 4	Average
≥ 850	≥ 825	≥ 825	≥ 825	≥ 800

Select matching region

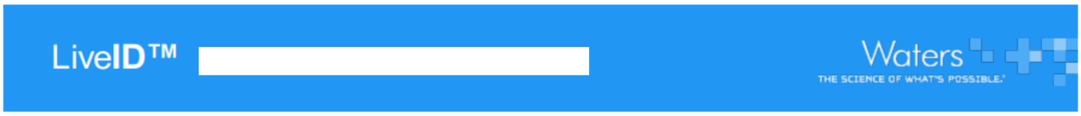

Results: Region 1

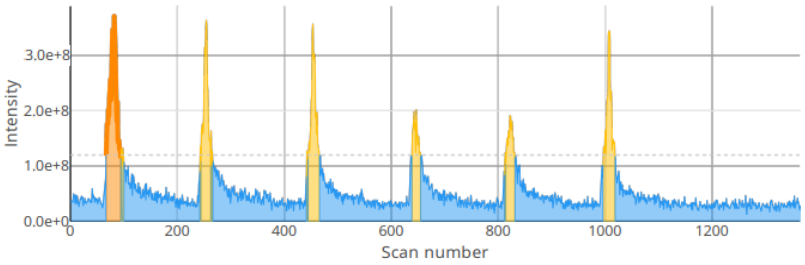

Check match scores

Compound	Function 1 (12V)	Function 2 (20V)	Function 3 (30V)	Function 4 (40V)	Match Score
Testosterone propionate	975	991	961	951	971
Testosterone enanthate	970	988	953	950	966
Testosterone isocaproate	896	979	970	898	928
Trenbolone	795	913	940	850	859

Check spectral data

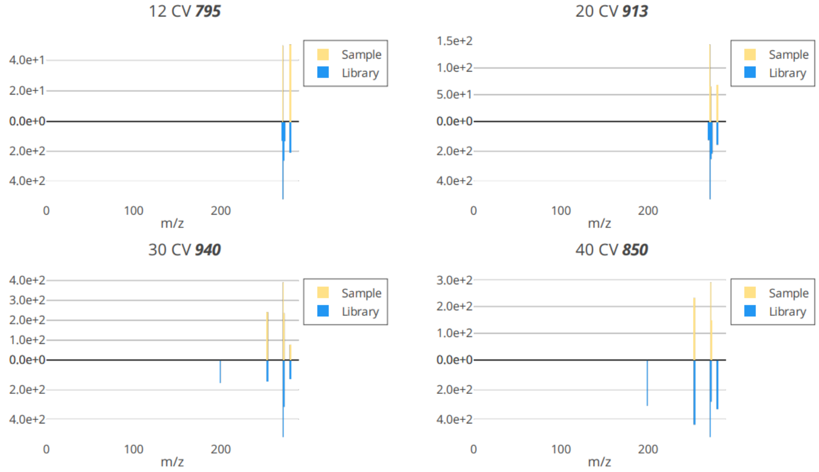

Fig. S6: LiveID software interface workflow for non-expert users to apply the method to real samples.
